# Mid-term low back pain improvement after total hip arthroplasty in 306 patients with developmental dysplasia of the hip

**DOI:** 10.1186/s13018-023-03701-z

**Published:** 2023-03-17

**Authors:** Cheng-Qi Jia, Yu-Jie Wu, Shi-Qi Cao, Fan-Qi Hu, Zhi-Rong Zheng, Chi Xu, Xue-Song Zhang

**Affiliations:** 1 Department of Orthopedics, Chinese PLA General Hospital, 28 Fuxing Road, Haidian District, Beijing, 100853 China; 2 Medical School of Chinese PLA, Beijing, China; 3 Department of Nursing, The Third People’s Hospital of Datong, Datong, Shanxi China; 4 Department of Orthopedics of TCM Clinical Unit, 6th Medical Center, Chinese PLA General Hospital, Beijing, China; 5 Present Address: Department of Orthopedics, Beijing Jishuitan Hospital, Beijing, 100035 China

**Keywords:** Developmental dysplasia of the hip, Total hip arthroplasty, Low back pain, BPFS, DDH

## Abstract

**Background:**

Low back pain (LBP) from hip and spinal disorders has been one of the main reasons for visiting physicians in patients with developmental dysplasia of the hip (DDH). It is essential to identify the LBP improvement among all grades of DDH patients treated with total hip arthroplasty (THA) at 5-year follow-up.

**Methods:**

The study included 407 hips of 306 patients (38 males, 268 females) who underwent THA between July 2007 and December 2016. There were 65 hips in Crowe I, 61 hips in Crowe II, 69 hips in Crowe III, and 212 hips in Crowe IV. One hundred and fourteen hips received subtrochanteric shortening. Patients included 101 bilateral THA (BTHA) and 205 unilateral THA (UTHA). The evaluation was performed through Back Pain Function Scale (BPFS), Harris hip score, Visual Analogue Scale (VAS), operative data and radiographic examinations.

**Results:**

The BPFS in patients of unilateral Crowe III and IV relieved significantly more (*p* < *0.05*). However, the BPFS in patients with bilateral symmetry DDH hips relieved significantly less than other groups of DDH hips (*p* < *0.05*). Harris in hips of Crowe II improved significantly more (*p* < *0.05*). The VAS in hips of Crowe II and III improved significantly more (*p* < *0.05*). The unilateral THA surgical time, blood loss, blood transfusion, and osteotomy number and length in Crowe IV were significantly more (*p* < *0.05*).

**Conclusion:**

THA is reliable to relieve LBP in DDH patients of unilateral Crowe III and IV; however, in patients with unilateral Crowe I, Crowe II, and bilateral DDH hips, the LBP improvements were limited. This should assist shared decision-making between orthopedic surgeons and patients.

**Level of evidence:**

Therapeutic Level II. See Instructions for Authors for a complete description of levels of evidence.

## Introduction

Patients with developmental dysplasia of the hip (DDH) often suffer low back pain (LBP), hip pain and limp for limb length discrepancy [1]. Among these symptoms, LBP is one of the main reasons for visiting physicians, which affects patients of all ages and causes disability and psychosocial problems [2]. Total hip arthroplasty (THA) is an important operation for patients with DDH. It not only improves hip function, but also relieves LBP of patients by restoring limb length and function [3]. However, appeared among all grades of DDH patients, LBP is a complex and multifactorial condition that hip osteoarthritis and lumbar spinal disorder often coexist in one DDH patient, significantly exacerbating mobility impairment [4, 5].

At present, most studies on LBP of DDH patients separated spine and hip problems, and mainly focused on Crowe IV patients or radiographic measurements which were easily influenced by the operators [4]. Moreover, there was a lack of an assessment of LBP to take into account hip and spinal disorders as a whole, and no specific scale for LBP. Patients with a hip dislocation usually showed abnormal spinopelvic alignment [6]. And it took a long time for DDH patients after THA to gradually establish new walking habits and adapt to new hip relationships. To our knowledge, among different grades of DDH patients, reliable research with sufficient quantity and midterm LBP relief after THA was rare.

The Back Pain Function Scale (BPFS) is a developed 12-item scale that introduces a new aspect of health-related quality of life, the patient’s difficulty in completing daily activities [6]. It can take into account hip and spinal disorders as a whole, through completing daily activities, for quantitative assessment.

The aim of this study was to use BPFS taking into account hip and spinal disorders as a whole to identify the LBP improvement among all grades of DDH patients treated with THA at 5-year follow-up.

## Patients and methods

### Study design

Between July 2007 and December 2016, 309 patients (410 hips) with LBP received THA due to osteoarthritis caused by DDH. Institutional review board approval and related informed consent were obtained.

The inclusion criteria were as follows: (1) DDH patients who underwent THA with LBP [7]; (2) a minimum follow-up period of 5 years. The exclusion criteria were as follows: (1) The LBP was caused by a history of rheumatoid or ankylosing spondylitis involving other special spinal conditions, but not DDH; (2) The LBP was caused by spine surgery or any medical disability that limited the ability to walk, but not DDH; (3) disabling diseases involving other joints of the lower extremities, and severe deformities (varus angulation, valgus angulation, or flexion contracture of more than 15°); (4) mental diseases.

**Fig. 1:**
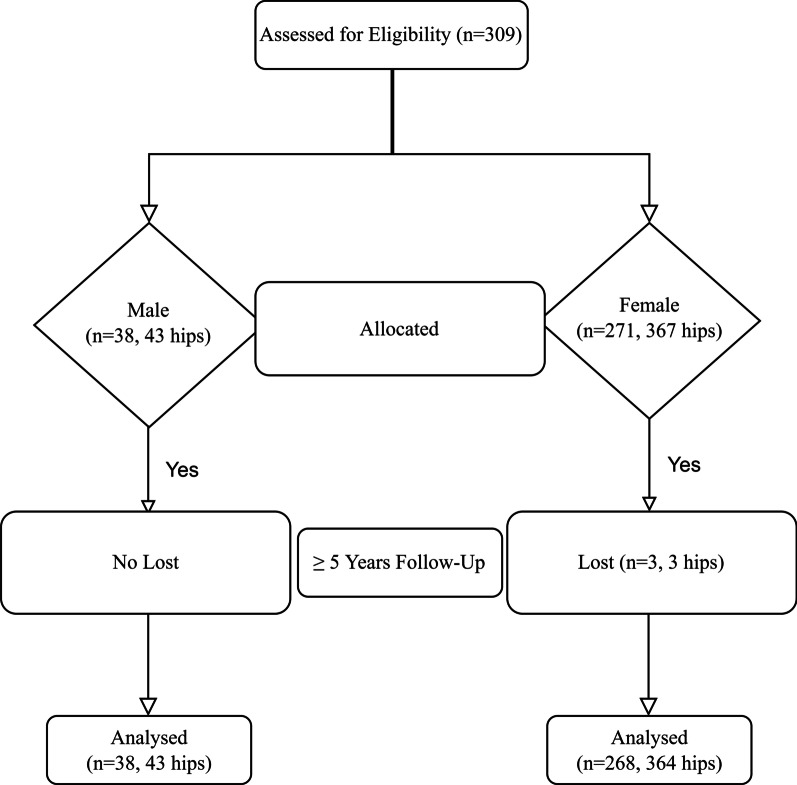
Flow diagram

Three female patients (3 hips) lost communication during the 5-year follow-up after THA. Finally, 306 patients (407 hips) met the inclusion criteria (Fig. 1). All patients were followed up ≥ 5 years (range 5–15 years). The remaining patients included 101 bilateral THA (BTHA) and 205 unilateral THA (UTHA). There were 65 hips in Crowe I, 61 hips in Crowe II, 69 hips in Crowe III, and 212 hips in Crowe IV (Table 1).

**Table 1:** Patient demographic parameters

Parameters	Male (*N* = 38, 43 hips)	Female (*N* = 268, 364 hips)
Age^**†**^ (year)	41.24 ± 13.56	44.77 ± 12.29
Height^**†**^(cm)	166.71 ± 7.08	157.56 ± 6.76
Weight^**†**^ (kg)	67.07 ± 14.66	57.66 ± 9.68
BMI^**†**^ (kg/m^2^)	24.06 ± 4.75	23.21 ± 3.58
Short side of legs		
Left	18	100
Right	8	109
Equal length	12	59
Limb length discrepancy^**†**^ (cm)	3.48 ± 1.71	3.27 ± 1.78
Crowe (no. [%])		
I	8	57
II	8	53
III	7	62
IV	20	192
Hip (no. [%])		
Unilateral DDH hip		
Crowe I	6	25
Crowe II	6	29
Crowe III	7	27
Crowe IV	14	91
Asymmetry bilateral DDH hips		
Crowe I–II	0	2
Crowe I–III	0	6
Crowe I–IV	2	4
Crowe II–III	0	6
Crowe II–IV	0	4
Crowe III–IV	0	9
Symmetry bilateral DDH hips		
Crowe I–I	0	10
Crowe II–II	1	6
Crowe III–III	0	7
Crowe IV–IV	2	42
Postoperative inpatient days^**†**^ (d)		
Bilateral THAs	10.40 ± 3.44	10.15 ± 5.97
Unilateral THA	11.45 ± 6.91	9.84 ± 4.44
Hip operation history (no. [%])	8	75
Follow-up^**†**^ (mo)	124.85 ± 28.78	109.89 ± 32.35

*BMI* body mass index, *THA* total hip arthroplasty, *DDH* developmental dysplasia of the hip

^†^The values are given as the mean and the standard deviation. *p < *0.05*

The data of the operative data included surgical time, intraoperative blood loss and transfusion, prosthesis types, revision rate and complications (Table 2). The complications were noted immediately (Table 2).

**Table 2:** Comparison of different Crowe types at 5 years after THA

Clinical factor	Crowe I (65 hips)	Crowe II (61 hips)	Crowe III (69 hips)	Crowe IV (212 hips)	*p*
Hip operation history (no. [%])	10	14	16	43	*.663*
Unilateral DDH					
Postoperative inpatient days^**†**^ (d)	10.39 ± 6.00	7.89 ± 2.76	8.24 ± 4.49	9.43 ± 4.26	*.069*
Surgical time^**†**^ (min)	125.78 ± 46.29	148.47 ± 65.07	133.51 ± 48.71	168.86 ± 65.89	.000*
Blood loss^**†**^ (mL)	442.61 ± 343.38	442.22 ± 310.05	497.96 ± 362.57	616.21 ± 455.02	.013*
Blood transfusion^**†**^ (mL)	235.87 ± 356.50	335.44 ± 423.07	206.73 ± 297.89	510.07 ± 718.81	.002*
Osteotomy (no. [%])	0	9	11	124	.000*
Osteotomy length^**†**^ (cm)	0	2.11 ± 1.29	2.32 ± 0.93	3.44 ± 1.20	.000*
Acetabular prosthesis position (no. [%])					.000*
Crowe I	44	56	43	197	
Crowe II	21	5	20	11	
Crowe III	0	0	6	3	
Crowe IV	0	0	0	1	
Acetabular prosthesis (no. [%])					.000*
Duraloc	12	13	20	66	
Betacup	29	20	7	5	
Combicup	15	17	27	53	
Pinnacle	9	11	15	88	
Femoral prosthesis (no. [%])					.000*
LCU	31	28	27	18	
S-Rom	34	29	40	169	
Corail	0	4	2	25	
Acetabular liner (no. [%])					*.177*
MOP	3	3	6	22	
3rd-COC	7	10	6	39	
4th-COC	55	48	57	151	
Revision hip (no. [%])					*.449*
Periprosthetic fractures	0	0	2	3	
Periprosthetic infection	0	0	0	1	
Polyethylene lining wear	0	0	0	2	
Complications (no. [%])					*.826*
Postoperative dislocation	0	0	0	2	
Limited flexion	0	0	0	2	
Limp	28	19	21	72	
Knee valgus	13	8	9	46	
Knee pain	2	1	3	3	
Thigh pain (distal femoral prosthesis)	4	1	2	4	
Hip abnormal noise	3	1	2	13	

*MOP* metal on highly cross-linked polyethylene, *3rd-COC* the third generation ceramic on ceramic, *4th-COC* the fourth generation ceramic on ceramic

^†^The values are given as the mean and the standard deviation. *p < *0.05*

Because the assessment of LBP was carried out by the individual as a whole, according to whether the height of bilateral hips dislocation of one DDH patient were symmetrical, the bilateral DDH patients were divided into the symmetry bilateral DDH hips group and the asymmetrical bilateral hips group. Then we had three groups: unilateral DDH hip group, symmetry bilateral DDH hips group and the asymmetrical bilateral hips group (Table 3). In addition, the preoperative and postoperative leg length discrepancy both were measured from the center of the femoral head to the superior border of the talus according to the X-ray of full-length standing anteroposterior [5]. The location of the acetabular prosthesis was classified according to the Crowe classification: acetabular prosthesis in Crowe I referred to acetabular prostheses were located in the femoral head position of Crowe I, and so on.

**Table 3:** Comparison of BPFS among Different Groups at 5 Years after THA

Clinical factor	Preoperative						*p*	Postoperative						*p*
	Unilateral DDH Hip (*N* = 205)				Bilateral DDH Hips (*N* = 33)	Bilateral DDH Hips (*N* = 68)		Unilateral DDH Hip (*N* = 205)				Bilateral DDH Hips (*N* = 33)	Bilateral DDH Hips (*N* = 68)	
	Crowe I (*N* = 31)	Crowe II (*N* = 35)	Crowe III (*N* = 34)	Crowe IV (*N* = 105)	Asymmetry	Symmetry		Crowe I (*N* = 31)	Crowe II (*N* = 35)	Crowe III (*N* = 34)	Crowe IV (*N* = 105)	Asymmetry	Symmetry	
BPFS^†^	.52 ± .20	.45 ± .22	.29 ± .16	.31 ± .18	.51 ± .24	.52 ± .23	.000*	.987 ± .049	.989 ± .047	.988 ± .048	.991 ± .051	.95 ± .13	.92 ± .18	.000*

*BPFS* Back Pain Functional Scale, *DDH* developmental dysplasia of the hip

^†^The values are given as the mean and the standard deviation. *p < *0.05*

Preoperative and postoperative clinical evaluations were performed by two independent orthopedic surgeons according to BPFS [8, 9], Harris hip score and Visual Analogue Scale (VAS) (Tables 3, 4). Data results were cross-checked by the other two independent orthopedic surgeons.

**Table 4:** Comparison of Harris and VAS before THA and 5 Years after THA

Clinical factor	Preoperative				Postoperative				*p*
	Crowe I (65 hips)	Crowe II (61 hips)	Crowe III (69 hips)	Crowe IV (212 hips)	Crowe I (65 hips)	Crowe II (61 hips)	Crowe III (69 hips)	Crowe IV (212 hips)	
Harris ^†^	56.71 ± 17.49	47.46 ± 18.96	52.19 ± 19.15	56.52 ± 17.71	93.28 ± 5.17	93.57 ± 5.57	94.05 ± 4.97	94.51 ± 5.32	.009*
VAS^†^	7.88 ± 1.24	8.11 ± .97	8.01 ± .83	7.76 ± 1.12	.38 ± 1.17	.07 ± .36	.03 ± .24	.08 ± .55	.017*

*Harris* Harris hip score, *VAS* Visual Analogue Scale

^†^The values are given as the mean and the standard deviation. *p < *0.05*

### Operation procedures

The acetabular prosthesis included Link Betacup (Link, Hamburg, Germany), Link Combicup, Depuy Pinnacle (DePuy, Warsaw, USA), and Depuy Duraloc [10]. The femoral prosthesis included Link LCU, Depuy Corail, and Depuy S-rom [11]. The surgeries were performed by one surgeon team. Briefly, all patients were operated through the posterolateral approach. After we resected the femoral head and eliminated fibrous tissue and osteophytes to reveal the true acetabulum, the acetabulum was reamed gradually to achieve the medial wall of the true acetabulum with bleeding spongy bone. Porous-coated acetabular prostheses were placed in the true anatomic acetabular position or higher position with enough bone stock coverage of the acetabular cup. If it was difficult to reset the hip during the surgery, the shortening subtrochanteric osteotomy (SSTO) was performed. The position of osteotomy was located at the distal end of the sleeve or cone. Cerclage wiring (two or three steel wires) was done around the location of the osteotomy to prevent fractures. The limb was held in flexion to relax the sciatic nerve after THA and this position had to be maintained for a few days [12].

### Statistical analysis

SPSS 24.0 (SPSS Inc) was used for statistical analysis. Categorical variables were presented as frequencies and continuous variables as means and standard deviation. The level of statistical significance was defined as *p* < 0.05. Independent sample t tests and Student–Newman–Keuls were performed in inpatient days, surgical time, intraoperative blood loss and transfusion, osteotomy length, BPFS, Harris, and VAS. Chi-square test or Fisher exact test were performed to determine the difference in prosthesis distribution.

## Results

Thirty-eight men and 268 women with an average age of 41 and 45 years at the time of surgery, respectively, were eligible in our study (*p* > *0.05*) (Table 1). The mean body mass index (BMI) for men and women was 24.06 ± 4.75 and 23.21 ± 3.58 kg/m^2^, respectively (Table 1). In our study, the mean postoperative inpatient days of Bilateral THAs for men and women was 10.40 ± 3.44 and 10.15 ± 5.97 days, respectively, the mean postoperative inpatient days of Unilateral THAs for men and women was 11.45 ± 6.91 and 9.84 ± 4.44 days, respectively (Table 1). The number of hip operation history for men and women was 8 and 75, respectively (Table 1). The mean limb length discrepancy for men and women was 3.48 ± 1.71 and 3.27 ± 1.78 cm, respectively (Table 1).

From Crowe I to Crowe IV, the number of hip operation history was 10, 14, 16, and 43 (*p* > 0.05), the mean postoperative inpatient days of unilateral DDH were 10.39 ± 6.00, 7.89 ± 2.76, 8.24 ± 4.49 and 9.43 ± 4.26 days, respectively (*p* < *0.05*), the surgical time of unilateral DDH was 125.78 ± 46.29, 148.47 ± 65.07, 133.51 ± 48.71 and 168.86 ± 65.89 min, respectively (*p* < *0.05*), the blood loss was 442.61 ± 343.38, 442.22 ± 310.05, 497.96 ± 362.57 and 616.21 ± 455.02 mL, respectively (*p* < *0.05*), and the blood transfusion was 235.87 ± 356.50, 335.44 ± 423.07, 206.73 ± 297.89 and 510.07 ± 718.81 mL, respectively (*p* < *0.05*) (Table 2). The number of THA with osteotomy was 0, 9, 11 and 124 from Crowe I to Crowe IV, and the mean osteotomy length was 0, 2.11 ± 1.29, 2.32 ± 0.93 and 3.44 ± 1.20 cm (*p* < *0.05*) (Table 2). In aspect of the acetabular prosthesis position 340 were in the true acetabulum/Crowe I (83.5%), 57 in Crowe II (14%), 9 in Crowe III, and only one in Crowe IV (*p* < *0.05*) (Table 2). The distribution of acetabular and femoral prosthesis type among different Crowe types were significantly different (*p* < *0.05*) (Table 2). The distribution of complications among different Crowe types were not statistically different (*p* > 0.05) (Table 2). Six hips in female accepted revision surgery, five for periprosthetic fractures, and one for periprosthetic infection. Two hips in male accepted revision surgery for polyethylene lining wear (Table 2). Postoperative dislocation happened in 2 Crowe IV hips of two female patients, and one of the females had two consecutive dislocations, who was 41 years old with two hips and a good Harris. These dislocations were all treated with closed reduction. Two male patients experienced postoperative flexion limitation, and their subsequent functional recovery were not good. Radiographic evaluation demonstrated excellent osteointegration of the implants in other cases.

And the preoperative mean BPFS of unilateral DDH were 0.52 ± 0.20, 0.45 ± 0.22, 0.29 ± 0.16 and 0.31 ± 0.18 from Crowe I to Crowe IV, and postoperative BPFS were 0.987 ± 0.049, 0.989 ± 0.047, 0.988 ± 0.048 and 0.991 ± 0.051, respectively (Table 3). The preoperative mean BPFS of asymmetry bilateral DDH hips and symmetry bilateral DDH hips were 0.51 ± 0.24 and 0.52 ± 0.23 (*p* < *0.05*), and postoperative BPFS were 0.95 ± 0.13 and 0.92 ± 0.18, respectively (*p* < *0.05*) (Table 3). The BPFS in patients of unilateral Crowe III and IV relieved significantly more (*p* < *0.05*). However, the BPFS in patients with bilateral symmetry DDH hips relieved significantly less than other groups of DDH hips (*p* < *0.05*).

For Crowe I (65 hips), Crowe II (61 hips), Crowe III (69 hips), and Crowe IV (212 hips), the mean values of postoperative Harris were 93.28 ± 5.17, 93.57 ± 5.57, 94.05 ± 4.97 and 94.51 ± 5.32, respectively (*p* < *0.05*), the mean values of postoperative VAS were 0.38 ± 1.17, 0.07 ± 0.36, 0.03 ± 0.24 and 0.08 ± 0.55, respectively (*p* < *0.05*) (Table 4).

## Discussion

LBP is a complex and multifactorial condition, and appears among all grades of DDH patients [4]. The BPFS is a disease-specific questionnaire measuring the influence of the LBP on patient’s perception of work, hobbies, home activities, bending or stooping, putting on shoes or socks, lifting, sleeping, standing, walking, climbing stairs, sitting, and driving [8]. Moreover, some of these questions could only be successfully completed 5 years after THA. All questions are answered in unable to perform activity, extreme difficulty, quite a bit of difficulty, moderate difficulty, a little bit of difficulty, and no difficulty, corresponding to 0–5 points, respectively. Lower scores refer to worse outcomes [13]. Our study took into account hip and spinal disorders as a whole, through BPFS, to identify the LBP improvement among all grades of DDH patients treated with THA.

We observed that patients with unilateral Crowe III and IV improved significantly in BPFS (*p* < *0.05*). One reason for this was the lower preoperative BPFS compared with other groups (*p* < *0.05*). According to some researchers, the flexion contracture of the hip joint led to increased pelvic forward tilt, lumbar lordosis, and, as a result, LBP. The condition was obvious in patients with unilateral Crowe III and IV, especially when they limped after a while [14]. The other reason was the significant improvement in limb length discrepancy after THA in the two groups, which had a certain effect on the recovery of the Cobb angle. Okuzu, etc., reported patients with LBP before THA, 62.9% had improved LBP at 1 year after THA, the Cobb angle improvement could be the key factors associated with LBP relief after THA [4]. However, in patients with unilateral Crowe I and II, the relief of LBP after THA were limited. This may be for the improvement in limb length discrepancy after THA in the two groups were not significantly compared with patients with unilateral Crowe III and IV. And even when their limb length discrepancy after THA was improved, the LBP part from spinal disorders was not wholly improved.

The LBP improvement after THA in patients with bilateral asymmetry and symmetrical DDH hips were limited. Spinopelvic alignment is an essential factor for maintaining an energy-efficient posture in individuals [6]. Various spinopelvic alignment parameters have been evaluated before or after THA in studies, including pelvic incidence, pelvic tilt, sacral slope, sagittal vertical axis, lumbar lordotic angle, thoracic kyphosis angle, and coronal lumbar angles, problems from which exist in patients with bilateral DDH hips. Therefore, it was difficult to recover quickly after THA in daily life due to the spine-pelvis-hip changes caused by preoperative bilateral high dislocation of bilateral hips [5]. Some articles reported that the risk factors for persistent LBP after THA were a high Cobb angle of the spine and tilted pelvis [4] that the more the pelvis tilted posteriorly, the higher was the risk of LBP after THA, however, the pelvic tilt and lumbar lordosis generally coexisted for a long time after THA [15]. Besides, the preoperative BPFS in patients with bilateral asymmetry and symmetrical DDH hips were comparable with patients of unilateral Crowe I and II and higher than patients of unilateral Crowe III and IV. Then the degree of LBP improvement after THA in patients with bilateral DDH hips was indeed limited.

Hip-spine syndrome was originally described by Offierski and MacNab [16]. Currently, there are studies that seek to assess the changes in spinal alignment and LBP, as well as the connections between THA and LBP [4, 10]. However, evidence on the hip-spine relationship mainly focused on Crowe IV patients, or radiographic measurements which were easily influenced by the operators, or spine and hip were separated [17, 18]. There was no specific quantitative scale for LBP, nor an overall assessment of the LBP change before and after THA in DDH patients among different grades [19, 20]. As a whole, the results of these studies were still not clear or just a bunch of measurements, the results are not intuitive. Therefore, we took into account hip and spinal disorders as a whole, through completing daily activities, providing a more intuitive LBP status before and after THA in DDH patients with different grades, to assist shared decision-making between orthopedic surgeons and DDH patients.

In Harris hip score, patients in Crowe II improved significantly (*p* < *0.05*). In VAS, patients in Crowe II and III had more hip pain relief (*p* < *0.05*). It was easier to reduce the hip in Crowe II and III than in Crowe IV [21, 22]. And THA was a selective operation, the time of THA in Crowe I patients was generally late. It was not until the acetabular and femoral head wear seriously caused the hip serious pain to THA. Therefore, the hip tissue tension of Crowe I patients was tight, and not as good as Crowe II and III when THA. Cameron et al. reported that postoperative Harris hip scores of patients with Crowe I were significantly lower than those of patients with Crowe II-IV. Crowe IV patients had significantly more complications than patients with lower Crowe grades [23]. Besides, hip pain relief of Crowe IV was not obvious because part of them had surgery just to improve gait [24, 25]. Our study adds to the existing body of the literature.

There are several limitations in the study. The sample size of the male was relatively small. Secondly, the amount of prosthesis distribution in different Crowe grades was also relatively small. Further analysis in subgroup was difficult. Changes in spinopelvic alignment after THA improve with time. LBP usually improves markedly over time following THA. We will continue to track and collect cases for 10–20 years.

## Conclusion

THA is reliable to relieve LBP in DDH patients of unilateral Crowe III and IV; however, in patients with unilateral Crowe I, Crowe II and bilateral DDH hips, the LBP improvements were limited. This should assist shared decision-making between orthopedic surgeons and patients.

## Data Availability

The datasets generated and/or analyzed during the current study are not publicly available due to some of the patient’s data regarding individual privacy, and according to the policy of our hospital, the data could not be shared with others without permission, but are available from the corresponding author on reasonable request.

## Abbreviations

DDH: Developmental dysplasia of the hip
THA: Total hip arthroplasty
LBP: Low back pain
BTHA: Bilateral THA
UTHA: Unilateral THA
BMI: Body mass index
VAS: Visual Analogue Scale
BPFS: Back pain function scale
SSTO: The shortening subtrochanteric osteotomy
